# A History of Heat Health Management Policies in the Singapore Military

**DOI:** 10.3390/healthcare11020211

**Published:** 2023-01-10

**Authors:** Joshua Dao Wei Sim, Jason Kai Wei Lee

**Affiliations:** 1 Department of History, Faculty of Social Sciences, Hong Kong Baptist University, Kowloon Tong, Hong Kong; 2 Heat Resilience and Performance Centre, National University of Singapore, Singapore 117597, Singapore; 3 Human Potential Translational Research Programme, Yong Loo Lin School of Medicine, National University of Singapore, Singapore 117597, Singapore; 4 Department of Physiology, Yong Loo Lin School of Medicine, National University of Singapore, Singapore 117593, Singapore; 5 Campus for Research Excellence and Technological Enterprise (CREATE), 1 CREATE Way, Singapore 138602, Singapore

**Keywords:** exertional heat injuries, history of sports medicine, history of sports science, warfighter injuries, military injuries, thermoregulation and exercise

## Abstract

Our paper, which is the first historical study about heat injuries in Singapore, seeks to situate the Singapore Armed Forces’ (SAF) history of heat stress management policies within the national context. Firstly, we observe that since the late 1970s, a research-driven approach has been adopted by the SAF’s military medical leaders to formulate a range of policies to address the Forces’ high incidence of heat injuries. This has resulted in the introduction of SAF-wide training measures, and the assembling of local scientific research expertise, which has led to a sharp reduction in heat injury incidence from the 1980s to 2000s. Through this, the SAF sought to demonstrate that its heat stress mitigation measures made the Singapore military ‘heat proof’. Secondly, the state shaped a soldier safety agenda in the late 2000s on the back of an increasing emphasis on safety and the transformation of the SAF into a highly-educated and technologically-sophisticated force. This meant a shift towards concern about the welfare of every soldier, particularly through the state’s drive to eradicate all training-related deaths. Accordingly, the SAF medical military leaders responded to the state’s safety agenda by introducing heat stress management research and policies that were oriented towards the target of eradicating deaths due to heat stress. This policy and research direction, as such, has been strongly guided by the state’s safety agenda and utilised to demonstrate to the public that all efforts have been taken to comprehensively mitigate the risks of heat.

## 1. Introduction

The prevention and treatment of heat illnesses has been a longstanding issue of concern among modern militaries since the First World War (WWI). A large part of this concern arose since the deployment of Western military troops to hot and humid environments across the world. These deployments resulted in high numbers of heat casualties, including deaths from heat stroke [1]. By the Second World War (WWII), the scale of deployment was truly global, with a large amount of warfare involving hot environments across Africa and Asia. During this period, extensive research was carried out by the Allied forces to understand the physiological effects of military operations in these hot environments. Such research gave rise to the introduction of heat stress management policies and guidelines in the British and American militaries after WWII [1,2].

### 1.1. Military Heat Stress Management

There are a number of classical studies which have examined military heat stress management. The most comprehensive is a two-part study by Bricknell, who traces the military experiences of heat illnesses from the pre-WWI period to the 1990s. While the study focuses mainly on the British and American military experiences, there is some coverage of the Israeli, Indian and Singaporean military experiences. The first part of the study, which covers the pre-WWI period to the end of WWII, supplies a narrative on the incremental increase in knowledge about the physiology of heat illnesses in European, British and American militaries through epidemiological studies and experiments on prophylactic measures. An increasing understanding of the nature of heat illnesses due to hot climate deployments since WWI led to the implementation of various measures. These included the construction of heat stroke stations with insulation from the heat, water drinking guidelines, environmental temperature monitoring, heat acclimatisation, emphasis on prevention among ground commanders and medical officers, and physical training limitations in high temperatures [1]. The second part of the study focuses on the British and American experiences of institutionalising research-based heat stress management measures since the 1950s and 1960s. In particular, this part analyses the implementation of guidelines in the American military since 1954. The major innovation was the introduction of the Wet Bulb Global Temperature (WBGT) index as a major environmental temperature monitoring measure to guide and limit physical and operational training in the hot weather. The validity of the WBGT index was tested through the 1970s and 1980s and was eventually supplemented by further guidelines on fluid replacement and the work-rest cycle. Assessing the post-WWII research, Bricknell concludes that the various militaries ‘recognised the importance of heat illness as a preventable injury’ [2].

Two studies reported the results of successful pioneering preventive measures that were implemented in the United States of America (US) military during the 1950s to 1960s. The first discusses the implementation of preventive measures in two US Air Force basic training centres from the summers of 1956 to 1958. Due to the replacement of the Dry Bulb Temperature index by the WBGT index to guide training, as well as increased water availability and the scheduling of outdoor physical training to the cooler hours of the day, the number of heat stroke cases dropped to two cases in 1958, in comparison to 13 and 39 cases in 1956 and 1957 respectively [3]. Similarly, in the second study, a prevention programme was introduced at the Marine Corps Recruit Depot, Parris Island in 1954, resulting in a ‘five to tenfold reduction in the seasonal incidence in heat casualties’ from 1956–1960, in comparison to 1952 to 1953. Some measures included using the WBGT to differentiate training limits for unacclimatised and acclimatised trainees in hot weather, an emphasis on training acceptable levels of physical conditioning (especially for obese and unfit recruits), the active encouragement of fluid replacement, and the enforcement of seven hours of sleep [4]. After WWII, research was also carried out on mitigative measures like reducing load carriage, provision of clothing with better cooling properties, and in-depth investigations into heat acclimatisation programmes for hot weather deployment, including the use of heat chambers to induce chronic physiological adaptations [5]. Today, the WBGT, along with the other measures, continue to be fundamental instruments which are used by the US military and other militaries across the world to mitigate the risk of heat injuries. A recent article identifies a number of these preventive measures. They include water consumption, a work-rest cycle constructed according to the WBGT index, heat acclimatisation, pre-activity cooling methods, mock rehearsals to recognise heat injuries during training, and a strong leadership which evaluates the risk of heat injuries and implements preventive strategies [6].

As with the examples above, in postcolonial Singapore, research became an essential building block that guided the implementation of heat stress management polices in the nation’s military. Measures like the use of WBGT to guide work-rest cycles and water consumption also became the basis of the Singapore military’s heat stress management regime. In spite of Singapore’s location near the equator and large pool of military conscripts, little work has been undertaken to review its military’s heat health policies (‘heat health’, which refers to the impact of heat stress on human health, will be used interchangeably with ‘heat stress management’). Thus, this study will utilise a historical approach to examine the introduction and implementation of these policies in Singapore since the government’s introduction of military conscription in 1967.

### 1.2. Local Historical Context and Research Questions

Four years after the introduction of mandatory conscription for male Singapore citizens in 1967, the first medical report on exertional heat stroke (EHS) in the Singapore Armed Forces (SAF) was published. The study examined two cases of heat stroke, one involving a 19-year old national service recruit and another involving 31-year old soldier. Both men recovered, but suffered significant sequelae, such as the ‘mild slurring of speech’ and ‘signs of mild spasticity’ for the 19-year old [7]. The medical consequences detailed in the report foreshadowed the emergence of a serious heat injury problem in the SAF, first reported in 1978. This crisis—which was transformed into a ‘war against heat’—would last for decades, spanning approximately 100–200 heat injury cases per year, before the SAF declared that they had been able to reduce the cases to a significantly manageable figure of approximately 20 per year in 2011 [8]. By the late 1970s, the military leadership and its medical doctors were acutely aware that the lives of their servicemen were being loss to heat disorders even without being summoned to the battlefield. A study published by SAF’s Headquarters Medical Services in 1978 outlined the severity of the problem in stark terms: there were six deaths due to EHS from 1975 to 1977 and 438 reported cases of heat disorders in 1976 and 1977. The authors stressed unequivocally that heat stroke was a ‘serious medical emergency’, but that it was nevertheless preventable [9]. In May 1978, two more national servicemen also lost their lives to EHS while undergoing running tests [10].

The emergence of a high incidence of exertional heat injuries within the first decade of the SAF’s history as a new citizen army was an unwelcome problem. The SAF was created by the ruling People’s Action Party (PAP) as an ‘armed guardian’ to ensure the survival of Singapore as an independent nation-state—one that had no natural resources as it was separated from its Malaysian hinterland, as well as being a tiny, predominantly Chinese state in a Malay/Muslim sea which rendered it vulnerable [11,12]. As a force relying largely on mandatory national service (NS), the SAF was also conceived for ‘nation-building’ [12]. With no claim to a ‘birthright’ of playing a founding role in their own modern nation-state as had the Chinese People’s Liberation Army or the Indonesian National Armed Forces, the SAF has been subjected to the control of the state and has directly served the nation-building agenda of the PAP [13]. In order to convince Singapore’s Chinese-majority population to commit their sons to NS at the outset, founding Prime Minister Lee Kuan Yew recalled that they ‘had to reorientate people’s minds to accept the need for a people’s army and overcome their traditional dislike for soldiering’ [14]. Thus, as a citizen-dominated military that was built on notions of survival and vulnerability within a hostile region, unnecessary deaths and injuries due to heat disorders were the last thing that the PAP needed for its militarised exercise of nation building. This was a medical crisis that warranted an effective solution, so that the state would not be detracted from its task of constructing a military with a formidable, critical mass of soldiers that could be mobilised in short notice.

Although the occurrence of military-training heat injuries did not receive much public attention initially, it was recognised as an emerging national problem since the 1970s and 1980s by the top PAP politicians, public servants, and the SAF’s top brass and medical fraternity. As the first SAF sports medicine doctor and former Chief of Medical Corps (1995–2001), Lionel Lee explains, the frequent incidence of yearly EHS deaths during the military’s formative years was considered ‘very alarming’ to the SAF’s medical community [15].

In light of the elite, top-down scrutiny that heat injuries received since the SAF’s early years, this paper—which is the first attempt to write a history of the SAF’s heat stress management policies from a national perspective—aims to address two questions. Firstly, how did the new, independent postcolonial Singapore nation-state create policies to manage the exertional heat stress problem in the military which arose as a result of creating a conscript-reliant SAF? Secondly, how did the elites of the state, SAF and the civil service intervene to prevent and treat military heat injuries as part of their justification for the continuing need of a conscript-dependent SAF? We propose that the answers to the two questions lie in the (1) use of *research-driven scientific measures* to treat and prevent heat injuries effectively, and (2) adoption of what we call ‘soldier safety’ to shape the research work and measures on heat stress management. Therefore, for this study, ‘policy’ refers to a series of health-related measures, interventions and responses that were implemented in SAF’s physical and operational training to prevent and treat exertional heat injuries.

Two groups of establishment figures, that is, the state and the military medical fraternity, were key actors in producing and shaping these policy initiatives. By ‘state’, we refer to top-level political, civil service and military leaders such as the Ministry chiefs (politicians and public servants) and the Chief of the Defence Force, who took a direct interest in influencing the policy and research direction for heat stress management in training. The ‘military medical fraternity’ (henceforth, medical fraternity) pertains to both SAF medical doctors and military exercise scientists who played key roles in both the research and translation of research into health-related measures, training practices and safety protocols. These doctors and scientists were primarily part of the SAF Medical Corps and DSO National Laboratories—two organisations within Singapore’s defence sector which have fostered close biomedical research relationships. Prominent actors would include the Chief of Medical Corps, Chief Army Medical Officer, the physicians and scientists of the Soldier Performance Centre (SPC) (part of the SAF Medical Corps till 2018) and exercise scientists who worked in Defence Medical and Environmental Research Institute (DMERI) (part of DSO National Laboratories). It is important to note that while DSO has historically served as the primary research and development arm of the SAF, it was corporatised in 1997 [16]. In spite of its autonomy, it continues to work primarily for the research interests of the SAF. In practical terms, this means that DSO is given the space to undertake independent research. Nonetheless, DSO scientists have no authority to direct the implementation of their research outcomes in the SAF; such implementation comes under the purview of the SAF personnel who have been tasked to translate the research for the Forces’ usage [17].

### 1.3. Research-Driven Measures and Soldier Safety

This study shows that the usage of research-driven scientific measures (henceforth, research-driven measures) drastically reduced the number of heat injury cases within the SAF; soldier safety, on the other hand, became the state’s tool for promoting the ultimate goal of eradicating all training-related deaths (including EHS deaths) through the enhancement of research-driven measures (details will be examined in Section 3 and Section 4). Research-driven measures represent the main pillar for policy making in the SAF’s heat stress management history. These measures were initiated since the late 1970s and have served as the foundational basis for all heat stress management policies in the SAF. Soldier safety quickly became a fundamental policy emphasis after it was introduced in the 2000s, and it continues as a core consideration to the present-day. Two specific arguments are proposed, for research-driven measures and soldier safety, respectively.

In terms of research-driven measures, we propose that the medical fraternity was drafted and supported by the state to be the main shapers of the military’s heat stress policies since the late 1970s. In particular, sports medicine and exercise science knowledge increasingly became the standards by which the SAF formulated its physical training practices and doctrines, as well as specific heat injury management protocols. In order to create these standards, the medical fraternity did not just borrow findings and lessons from their counterparts in the West; institutions were also established to produce local medical and scientific knowledge, giving them the credibility to formulate both training and safety standards, transforming military training into regimes that were guided by research-based scientific recommendations. These research-driven measures, which were implemented in the 1980s, were able to drastically reduce the SAF’s heat injury burden by the early 2000s, resulting in a ‘new normal’ of low heat injury incidences since then. In effect, these measures transformed the SAF into a ‘heat-proof’ force for all Singaporean soldiers.

The introduction of soldier safety would see the widening role of the state from the early 2000s to the present-day. While the medical fraternity remained as the key knowledge producers and policy shapers, the direction of their work was increasingly influenced by the state’s deepening focus on soldier safety. Soldier safety represented the state’s intensifying concern on the health, wellbeing, and preservation of life of every individual NS man during peacetime training. It became the state’s construction of an overarching agenda that has been utilised as a legitimating tool for the continued justification of the SAF as a conscript-reliant military. Safety acts as the training goal, standard and core SAF value that soldiers are required to internalise. In particular, there has been a heightened concern for the life of every soldier, which has led to the adoption of the goal to eradicate all training-related deaths (including deaths related to EHS), and an intense focus on tightening and strengthening safety measures to prevent such occurrences. This meant that the state has taken the lead in encouraging and influencing policies relating to training-related safety. Their increasingly heavy-handed approach to soldier safety meant that the medical fraternity was compelled to handle short-term cycles of policy implementation involving the expedited translation of proven clinical and research-based findings, as well as ongoing research work into heat stress management measures. These cycles were in response to several high-profile training-related accidents and deaths, including a number of serious EHS cases, and the possible threat of increased heat stress on soldiers due to changes in military equipment and gear. The medical fraternity also adapted to the state’s direction by incorporating soldier safety as a key concern in their research work since the 2010s.

As can be seen, this study will demonstrate that the state wielded substantial clout over the heat stress management policies, especially since the 2000s. This corresponds with several social scientific and historical studies on health and safety policymaking in Singapore which also observe the state’s heavy hand, especially through technocratic governance. One main study is Michael Barr’s critical analysis of the state’s deployment of technocracy to reform Singapore’s healthcare system. Crucially, Barr observes that Singapore’s healthcare system has been seen as a model example of how technocrats utilised the principles of pro-capital economics and management to transform state-funded healthcare into a tiered-based, privatised model during the 1990s. This encouraged efficient resource allocation through a system of co-payments and the full privatisation of upper-tier services to meet the demands of the middle and upper classes. Barr opines that these technocratic impositions have been far from value-free, as the privatised model, by favouring capital, catered to the wants of the middle-class and marginalised the needs of the poor [18].

The state’s heavy-handedness has also been observed in the areas of tuberculosis management and industrial safety regulations. Specifically, it continually implemented and revised a wide range of health and occupational safety measures that tended towards excessive top-down management in order to mitigate the anxieties and consequences generated by its nation-building goals. Such extensive policy interventions did not necessarily result in additional beneficial outcomes, calling into question the level of state activism practiced in these policies [19,20]. This top-down approach can also be observed in the state’s recent handling of the COVID-19 crisis, although, unlike the previous examples, the ever-changing nature of this pandemic has meant that the state’s management and containment of the infections has been balanced by a more nimble approach which ‘swiftly’ incorporated new scientific evidence on the advice of the health authorities ‘as more of the (effects of the) virus became known’ [21,22]. While our paper affirms the findings of these studies, it also brings significant nuance by showing that the state was not always so heavy-handed in its approach, as the medical fraternity had the autonomy and authority to implement its research-driven measures from the 1970s to the 2000s, before concerns about soldier safety motivated the state to be more activistic.

It is important to clarify the medical distinctions between various terms that are used in this study. Heat stress is a generic term referring to the external (e.g., environmental and clothing) and internal (workload) thermal load of a person engaging in physical exertion, such as running, long-distance marching or fast-marching. Heat injuries or disorders, on the other hand, can be understood as ‘a collective of specific medical conditions that are related to the effects of exertion-related heat illness that varies in severity’ [23]. A significant type of injury is heat exhaustion, defined as the ‘inability to continue strenuous physical exertion due to fatigue from heat stress’ [24]. Heat stroke, the most serious of all heat injuries, is a ‘potentially fatal systemic condition that occurs when the [body’s] thermoregulation system is overwhelmed.’ It is often associated with hyperthermia and could lead to a ‘systemic inflammatory response’ which could ultimately result in multi-organ dysfunction, and in the worst-case scenario, death [24]. For the context of this study, we are referring only to exertional heat stroke (as well as exertional heat stress and injuries) which occurs during physical exercise, and not classical heat stroke which happens ‘during passive exposure to environmental heat stress’ [24]. Although conditions relating to exertional heat stress concerns the ability of the body to remove endogenous heat to cope with the physical exertion, environmental stress also plays an important role as Singapore’s hot-humid conditions heavily limits the human body’s evaporative ‘cooling mechanism’ (the main way heat is lost during exercise) [25].

## 2. Methods

### 2.1. Collection of Documentary Sources

The historical approach is the main method of this study. There are two main pillars in this approach. Firstly, a range of documentary sources were collected for analysis. A main group of sources are SAF-related sources which can be found at the National University of Singapore Medical and Central libraries. They include various local medical studies on heat injuries in the SAF during the 1970s and 1980s. Another important source is the *Pointer* journal—the official academic journal of the SAF. As we shall see, medical fraternity leaders published some of their findings and understandings about SAF heat injuries in *Pointer* during the 1990s. The journal also became a main forum to discuss ideas pertaining to soldier safety. The study also drew on two online databases, NewspaperSG and Factiva, to search for historical and contemporary media coverage on heat injuries in Singapore. NewspaperSG, a database of digitised Singapore and Malaya newspapers by Singapore’s National Library Board, was used to search for historical news articles from the 1970s to 1990s. Factiva, a business information and research tool which provides access to a global collection of free and licensed media information, was used to search for recent English-language reports and media coverage on heat injuries in Singapore from the late 1990s to 2021. The online database search engines of the National Archives of Singapore and the Parliament of Singapore were used to search for official documents, historical records, and records of parliamentary debates pertaining to heat injuries and training-related accidents in the SAF.

### 2.2. Use of Oral Histories

A second pillar of this study is the use of oral histories. Oral history refers to the use of recorded interviews to collect historically significant memories and personal views of informants who have been involved in the historical events and communities that are of direct interest to a researcher [26]. For this study, a total of seven oral history interviews were recorded with medical doctors and exercise scientists who worked for the SAF Medical Corps and DSO and had deep knowledge about research and policies pertaining to heat injuries and training-related accidents in the SAF. The semi-structured interview method was utilised. All informants received a list of questions relating to their research and policy experiences in the SAF Medical Corps and DSO at least one week prior to the interview. Interviews were then conducted with these questions, along with follow-up and clarification questions which arose during the course of the interviews. This integration of both documentary source analysis and oral history interviews enabled the authors to frame a historical interpretation which shows the shift from research-driven measures as the main driver of heat stress management policies to the state’s influence on these policies through its soldier safety agenda.

## 3. Medical-Historical Findings

### 3.1. Extent of Heat Injuries

**Figure 1:**
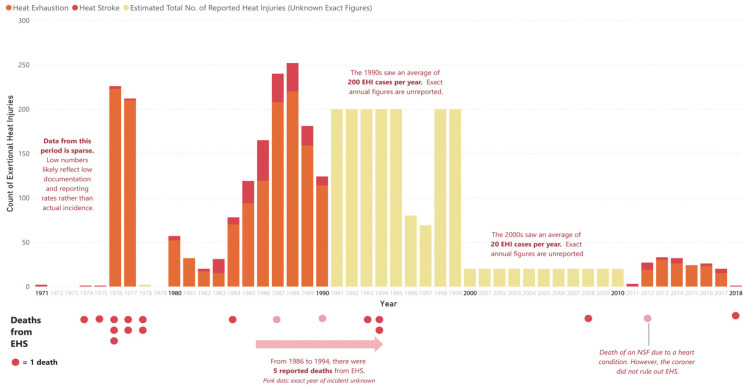
Graph of Publicly Reported Exertional Heat Injuries in the SAF, 1971–2018.

Since the 1970s, the SAF has never made its data on heat injuries fully transparent to the public. Nonetheless, during certain years, snapshots of such data were made available to the public. Table 1 and Figure 1 are a compilation of all the publicly-reported data on SAF heat injuries. We note that low incidence of EHS cases that is observed in some years may be a function of under-reporting rather than a reflection of the actual frequency of EHS cases. In particular, between 1971 and 2018, the data was observed to be incomplete or vague, with the exception of 1976–1984 and 2012–2018. In addition, from our knowledge, definitions of heat injuries were not consistent throughout this period.

Overall, the data points to a long-drawn, high-volume military heat injury burden that only came under control by the 2000s. It is important to note that while we do not have direct access to heat injury surveillance data produced by the SAF Medical Corps, based on the compilation of various publicly-reported data points, and various reports and studies that we refer to in Section 3, we are sufficiently confident that the SAF experienced a high heat injury burden till the late 1990s. A second observation is that there was a significant volume of total heat injury cases from the 1970s to the 1990s, where reported cases per year fell within the range of 100 to 250. While there were some exceptions, such as in 1982 where the figure of 30 was reported, the SAF did not register low double-digit figures consistently until the 2000s. Thirdly, compared to the other decades where reported heat stroke cases were mainly in single digits, there was a significant spike in EHS cases from 1984 to 1990. These observations raise two questions. How did the SAF respond to the high volume of heat injuries from the 1970s to 1990s? What solutions did they introduce to reduce the injury incidence and severity?

### 3.2. Research-Driven Measures: Medical Shapers of Military Heat Stress Management

Medical fraternity leaders had been key shapers of research-driven heat stress management measures and practices since the 1970s. Their ideas and efforts to produce knowledge on exertional heat stress had a direct impact on the way the SAF transformed their physical training and safety protocols. As mentioned, we observe that the medical fraternity was entrusted by the state to intervene in the heat injury crisis. This required the building up of local knowledge and the creation of suitable scientifically-based interventions that enabled them to implement a range of policies and practices that eventually exerted an influence on the way heat disorders were prevented and treated.

#### 3.2.1. Building up Local Knowledge

When NS was introduced in 1967, both the state and the medical fraternity did not foresee that exertional heat injuries would become a serious problem. Lionel Lee recalls that in those years, no one thought of heat injuries as ‘medical emergencies’, especially in the hospitals [15]. This meant that the first local studies on heat stress only emerged when there was a realisation about the gravity of the issue during the 1970s. In order to build up an understanding of the condition, Headquarters Medical Services began to collect data on heat injury cases during the mid-1970s before a mandatory ‘surveillance system’ was established in 1978 [9,27]. These initiatives coincided with the enlistment of the first batch of NS doctors in 1974, which enabled the medical officer positions in the Headquarters and across the SAF to be filled [35]. Therefore, through this system, the medical fraternity was able to gain control over the reporting of heat disorder cases across the SAF, and thereby create an initial epidemiological understanding of the situation.

Three reports that produced significant epidemiological findings were ‘Heat Stress Among Soldiers in Training’ (1978), ‘Heat disorders: the Singapore Armed Forces experience 1980–1984’ (1988) and ‘Prevention of Heat Disorders in the Singapore Armed Forces—1984–1989’ (1991). Together with a number of hospital-based surveillance studies, these papers laid down the fundamentals of Singapore’s heat stress management knowledge. Here, we trace some crucial findings. Firstly, the studies established that new recruits were the most susceptible to heat injuries, due to their lack of exposure and acclimatisation to physical exertion in the hot weather. The 1978 study reported that four out of the six EHS deaths from 1975 to 1977 were new recruits who ‘succumbed within 28 days of enlistment’ [9]. Later studies in 1988 also supported this finding. Two studies found that 40 and 45.8 percent of all heat injury cases happened during the basic military training phase from 1980 to 1984 and 1985 to 1986 respectively [28,32]. A 1988 hospital survey co-authored by Yeo and colleagues also found that 85 of their 94 admissions due to military-training heat disorders were new recruits. Many of these hospitalised recruits had also enlisted for basic training during the hot months from March to October, from 1974 to 1979. They concluded that strenuous exercise and the ‘lack of physical fitness and acclimatisation’, especially under a hot-humid climate, were major ‘predisposing’ factors [36]. This was affirmed in a 1991 essay by Low Wye Mun, another pioneering SAF sports doctor who was Lionel Lee’s junior, who explained that ‘the enlistee is more prone to heat disorders as a result of his generally poor physical conditioning and … lack of acclimatisation to functioning in the hot, humid outdoors’ [28]. These were important findings, given that earlier studies conducted with the US Army demonstrated the susceptibility of recruits to heat injuries during the early phases of their training [4,37].

Secondly, sickness prior to strenuous physical activity was identified as a strong risk factor. Out of the six EHS deaths from 1975 to 1977, two were found to have been ill with diarrhoea and fever prior to exercise, two ‘very significant’ illnesses associated with heat stroke [9]. Similarly, other studies identified fever, infection, diarrhoea, hypertension, upper respiratory tract infections and past history of heat injuries in the victims [36,38]. A third important finding was the type of physical activity associated with the heat injury occurrences. In particular, strenuous exercise that occurred over short to medium distances was highly associated with heat injuries. Yeo and colleagues found that five and 10 km runs preceded 69 out of 104 heat injury occurrences from 1974 to 1979 [36]. Lim, Pang and Tan’s 1988 survey showed that the highest incidence of heat injuries from 1980–1984 happened with runs (36%), field training (25%) and route marches (24%). 140 out of 223 cases also occurred in combat uniform attire [32]. These findings corroborate newspaper reports of three EHS deaths due to running in 1978 and 1984. One soldier, who died while completing his 10 km running test, was also dressed in combat uniform with a rifle, grenade pouch, bullets and a water canteen in tow [10,39]. Fourthly, surveillance results also indicated that there was a lack of consistency in treatment times and methods which resulted in the deaths of some victims. Yeo and colleagues, for instance, highlighted that the management of patients from their time of collapse to the hospital was ‘not standardised’. For a small number of cases, evacuation ‘time elapsed was unacceptably long’ while in terms of field treatment, only ‘one fifth’ of the victims received sponging with cold water—this meant that there was either a delay in the diagnosis or a lack of awareness that rapid cooling had to be immediately instituted for the victims [36].

The collection and publication of these surveillance results established the medical fraternity as the national experts in heat disorders. Their studies elucidated clear evidence about how NS training was associated with heat disorders. The joint publication of the first Singaporean volume on heat injuries in 1988—titled *Heat Disorders*—by doctors from the SAF and Ministry of Health was a demonstration of their consolidated knowledge and understanding of the illness. In particular, the editors, Peter Yeo, then Professor at the National University Hospital, and Lim Meng Kin, then SAF’s Chief Medical Officer (equivalent of the Chief of Medical Corps), had come to the conclusion that the ‘sometimes fatal, medical complications’ arising from exertional heat injuries were largely ‘preventable’ [40]. Evidently, the medical fraternity was claiming that with their production of local population-based knowledge, they had the capacity, know-how and authority to effectively reduce the high rates of morbidity and mortality associated with the condition.

#### 3.2.2. Science-Based Interventions

In Lionel Lee’s essay ‘Heat stroke: preventive measures’ in *Heat Disorders*, he laid out the SAF’s overall strategy for heat stress management. ‘The key to the preventive programme’, he argued, was to ‘identify the population at risk’. He continued: ‘Recruits who take part in training programmes with inadequate sleep and lack of acclimatisation, but who are higher disciplined and motivated will be victims of heat stroke if no strict preventive measures are taken. The challenge is also to educate the commanders to heed these necessary measures’ [41].

The noun ‘measures’ refers to a series of science-based interventions that were implemented by the medical fraternity to solve the heat injury problem. The epidemiological quantification of injuries, as well as the resultant interventions, became part of a series of solutions that were put in place by the medical fraternity to solve the national problem. These solutions resembled the emerging practice of evidence-based medicine, especially in terms of how the medical fraternity drew on the knowledge from their heat injury surveillance studies to implement the measures. Evidence-based medicine, which came to prominence in the mid-1980s and went on to dominate the practice of medicine since the 1990s, is an approach to clinical medicine stressing that ‘epidemiology … could be applied to individual patient care [42]’. Another article explains that evidence-based medicine ‘places the highest value on secondary “evidence” drawn from epidemiological research’. This challenges the traditional approach to medicine which prioritised the doctor’s clinical judgement over empirical evidence [43]. Keith Denny also notes that evidence-based medicine goes to the issue of medical power as it ‘reinforces such authority by regulating the conditions under which a physician may speak authoritatively about health and illness’ [44].

The medical fraternity’s authority to implement and transform physical training regimes and safety protocols in the SAF, which arose as a result of the knowledge they gained through their research, was a demonstration of how such authority was afforded to them by the state. In the first place, the medical fraternity sought to strengthen their expertise in the areas of heat injuries, training-related musculoskeletal injuries, physical fitness and obesity—key concerns related directly to the physical health and performance of all soldiers—by sending a number of their career medical officers to study sports medicine in the US in the 1980s and 1990s. Lionel Lee and Low Wye Mun were two prominent pioneering sports medicine doctors who were sent overseas for studies and went on to establish themselves as key medical fraternity leaders as a result of their expertise. In particular, Lee became extremely influential in formulating the fundamental guidelines for the SAF’s heat injury prevention programme. He was the first SAF doctor to graduate with a Master’s in Sports Medicine from the United States Sports Medicine Academy (linked to the University of South Alabama), one of the few institutions offering graduate degrees in this subject during that period, in 1983 (Lee was the second doctor in Singapore to be trained in sports medicine. The first was the late Teh Kong Chuan) [15,45].

Lee received strong support from Lieutenant-General Winston Choo, who was the Chief of General Staff and the SAF’s first Chief of Defence Force (1974 to 1992) and Kwa Soon Bee, who was the Permanent Secretary for Health and Director of Medical Services (the top civil servant in the Ministry of Health) from 1984 to 1996. Kwa was also the younger brother of Kwa Geok Choo, wife of Lee Kuan Yew, Singapore’s first Prime Minister [46,47]. Winston Choo backed Lionel Lee’s efforts to pioneer the SAF’s first Physical Performance Centre (PPC) in 1984, a sports medicine and exercise science unit focused on the provision of sports medical and physiotherapy clinical services, and exercise science and applied physical training research [15,35,48]. The PPC eventually became the Soldier Performance Centre under the leadership of Low Wye Mun in 1995/1996, transforming into the army’s specialised sports medicine and exercise science hub [49]. Kwa also supported many of Lionel Lee’s endeavours, partly because they had formed a ‘fantastic’ relationship as Lee served as Kwa’s deputy commander for the SAF’s combat service hospitals. As the Permanent Secretary (Health), the latter was concerned about the mismanagement of the heat injuries in the hospitals and he worked with Lionel Lee to establish two hospitals as heat disorder emergency centres. Both men also created the first ‘continuous’ system of heat stress management from the SAF camps to the hospitals, incorporating elements like helicopter evacuations, the continuous cooling of the patients in the emergency transports, and the installation of the Body Cooling Unit (BCU), a specialised evaporative cooling system, at the two hospitals [15,50].

The exclusive support received by Lionel Lee meant that he was empowered with the authority and influence to implement scientifically-based guidelines in response to the findings of the epidemiological studies as well as the spike in heat injury cases from 1985 to 1988 (see Table 1 and Figure 1). By this period, he had been promoted to the position of Senior Medical Officer (Army), the equivalent of the Chief Army Medical Officer today. The measures were based on four ‘time-tested pillars’: educational programmes, training safety regulations, surveillance of heat injury incidence and environmental thermal load, and the improvement of the heat disorder management in the SAF and the Ministry of Health. Essentially, by doing so, the medical fraternity was able to transform military training with scientific interventions which had been utilised by militaries in the West, but adapted for the conditions in Singapore. Some measures included:

➢ Educating all recruits, medical officers, commanders and trainers through the use of simple messages and symposiums. Recruits, for instance, were taught how to conduct emergency first aid on their ‘buddy’ (assigned partner during basic military training) who had collapsed due to heat injury.
➢ Safety regulations were incorporated into training programmes. This included a stress on ‘gradual and progressive increase in training load on a week to week basis’ to induce heat acclimatisation. Recruits were also grouped according to their physical fitness levels and prescribed appropriate training programmes.
➢ Specific training regulations were introduced to set limits to training. This included defining strenuous activity types and prohibiting ‘excessive strenuous activities’ during the hottest part of the day; setting limits on training, such as prohibiting running beyond 10 km; introducing a work-rest cycle for strenuous activities (e.g., 15 min of rest for every 60 min of continuous physical exertion); and introducing water ‘parades’ which enforced pre-strenuous exercise liquid consumption ‘beyond the point of thirst’ in order to prevent dehydration and EHS.
➢ The introduction of the BCU cooling system in ‘training schools where incidence[s] of heat disorders were high’, was followed by the installation of the system in almost every SAF camp by the end of the 1990s. Lee had learnt about the BCU during a trip to Israel in the early 1980s and worked with a Singapore defence engineer Koh Soo Keong to re-produce their own version of the BCU. Although the ‘gold’ standard for the rapid cooling of heat injuries is cold-water immersion, which can produce cooling rates of 0.15 to 0.24 °C/min, as compared to the SAF BCU which has produced cooling rates of 0.09 to 0.18 °C/min, the BCU was chosen as it was ‘well tolerated’ by patients and enabled ‘continuous monitoring and resuscitation’ [15,24,27,51,52].

Adopting these interventions proved to be effective as the continued surveillance of heat injuries in the 1990s and 2000s saw a sharp drop in heat injury incidences. These results motivated the medical fraternity to demonstrate the success of their work through the public quantification of the fall in rate and number of heat injuries in major local media outlets. Similarly, the technocratic transformation of the Singapore healthcare system meant a move towards the public reporting of its quantified achievements and outcomes. For instance, Singapore was ranked as the ‘most cost-effective health care system in ASEAN’ in 2004 while it also achieved a health expenditure rate that was ‘drastically lower’ than advanced Western economies like the United Kingdom and US [18]. In 1997, the SAF Headquarters Medical Corps reported in *The Straits Times* that heat injury cases had declined from ‘more than 250 cases in 1988 to an all-time low of 80’ in 1996. The report also explained that after the implementation of training regulations like water ‘parades’, work-rest cycles and the installation of the BCU in ‘24 SAF schools and camps in Singapore and abroad’, heat injury cases ‘plunged’ to 1.4 per 1,000 soldiers in 1996, compared to 6.9 per 1000 soldiers in 1987 [33]. In 2011, the SAF reported that its ‘[n]ew training regulations and education’ helped to reduce cases by 90 percent from the 1990s to about the number of 20 per year. Unfortunately, the paucity of publicly-reported data means that the pattern of the reduction in cases cannot be scrutinised. Most of the reported measures were similar to earlier ones which had been put in place under Lee’s command since the 1980s, except for the introduction of a new ‘heat acclimatisation’ package during basic military training, an issue which will be discussed in the next section. Additionally, BCU numbers had increased to 33 units. Then Deputy Chief Army Medical Officer, Lionel Cheng, concluded that as long as the SAF engaged in physical training, it would only be able to ‘keep heat injuries low but not “down to zero”’ [8]. To conclude, in order to demonstrate that the medical fraternity’s scientific management had managed to convincingly gain control of the national heat injury crisis, public quantification became the means to project its discourse of success.

## 4. Discussion

### 4.1. Soldier Safety: The State’s Shaping of Heat Stress Management Policy

Changes in the SAF and national attitudes about NS meant that a quantified discourse of success could not remain persuasive. As part of the research-driven measures introduced since the late 1970s, we have observed that the state afforded the medical fraternity the leeway to tackle the heat injury crisis through the use of local research and scientific interventions, while providing them with the necessary support. Reducing the SAF’s heat disorders was in the national interest. However, the state’s meaning of national interest had shifted by the 2000s. The ‘new normal’ of low heat injuries, a decreasing NS intake due to low birth rates since the 1980s and 1990s, a movement towards the treatment of soldiers as thinking operators, and the prioritisation of safety in order to prevent peacetime training deaths gave rise to a situation where the state and the medical fraternity held ‘different sets of expectations and interests’ in heat stress research and management [53]. Most significantly, although the medical fraternity continued to stress its autonomy to develop sports medicine and exercise science research and interventions, the state became increasingly influential by imprinting their new concern of soldier safety on the direction of the medical fraternity’s research in order to respond to parental concerns about the lives and well-being of their sons. This included a heightened focus on enhancing safety to eradicate all training-related deaths through initiatives which responded to issues of direct public concern, such as serious training-related accidents (including EHS). These moves have been reflective of the increasingly ‘consultative’ nature of the PAP government since the 1990s, which Barr characterises as a ‘display [of] unprecedented degrees of flexibility on matters of political sensitivity as it tries to emulate democracy’s positive feedback loop without surrendering control of the agenda’ [54]. In this sense, the stress on soldier safety became a means through which they expressed their utmost concern to protect the lives of every Singaporean soldier in order to retain the popular support for National Service. What this also meant was that the medical fraternity’s work to translate various research work and findings into measures became driven by short-term cycles as a result of the state’s sharp reaction to high-profile incidents and events, such as serious training-related accidents and deaths.

### 4.2. New Context

The development of soldier safety arose in a new national context pertaining to the SAF and views about NS. In the first place, the low number of publicly-reported heat disorders and EHS cases and/or deaths since 1997 meant that the state and public grew increasingly accustomed to this ‘new normal’ or ‘disappearing problem’ of low heat injury incidences [55]. In fact, by the late 1990s, the SAF had achieved a low number of training-related fatalities. The number of deaths decreased from six in 1997 to one in 1999, causing then Minister of Defence Tony Tan to laud these figures as an ‘improvement’ in the SAF’s initiatives to enhance safety without reducing ‘the tempo of training and training standards’ [56]. From 1994 to 2011, only four EHS deaths had been reported (see Table 1). The medical fraternity also contributed to the claims of this new normal by releasing its ‘winning formula’: their best practice, evidence-based clinical guidelines on heat injury management [8]. In the book’s foreword, Lionel Lee and Benjamin Seet (then Chief of Medical Corps) proudly proclaimed:

‘Over the years, the Singapore Armed Forces … has developed guidelines on the prevention and management of heat injuries. Through its research, programmes for acclimatisation, hydration and managing work-rest cycles … which coupled with soldier education, have substantially reduced the incidence of exertional heat injuries by more than ten-fold. In addition, the SAF has implemented an in-house evaporative body cooling unit which has been shown to effectively treat hyperthermia … This set of guidelines incorporates the best available evidence … and expert consensus to assist medical practitioners in the prevention and clinical management of exertional heat injuries’ [24].

The formulation of this narrative was an extension of their discourse of success. What this meant was that in this new normal, the medical fraternity sought to re-position themselves as the Republic’s (or even the region’s) foremost institution for heat stress management. Gone was the problem of high heat injury volume and frequently-reported EHS deaths. Instead, Singapore could look forward to the excellent prevention and treatment of the condition as a result of its rigorous practices and research.

Secondly, there were changes in thinking of how soldiers were viewed as manpower and combat operators. In terms of quantity, Singapore has been facing a shrinking birth rate that has not met the replacement rate since 1976. This has directly affected the annual recruit numbers, which have been placed between the range of 19,000 to 24,000 from 1990 to 2030. This has fallen short of the national needs-based projection of 25,000 conscripts per year. The continuously declining birth rate trend means that the numbers are expected to shrink to 15,000 annually, and to between 10,000 and 12,500 by 2065’ [57]. The decreasing quantity of conscripts, as well as the increasing material affluence and small family sizes that these children have grown up in have meant that the state has changed their approach and attitudes towards the soldiers, especially since the 2000s and 2010s. These changes include increased parental ‘access to their son’s NS journeys than ever before’, strong public expressions of appreciation by the SAF’s top brass to the commitment of conscripts, and also the allowance of ‘conscripts to express an interest in certain vocations’ since 2017 [57].

Shifts also occurred with the way soldiers were to be seen as combat operators. With better-educated conscripts and the realisation of the demands of the twenty-first century battlefield, a new generation of top military leaders during the late 1990s and 2000s called for a change towards viewing soldiers as thinking individuals. In 1999, a future Chief of Army and Defence Force, Desmond Kuek, envisioned that the twenty-first century SAF should ‘individualise standards’ and ‘maximise the potential of each individual’ by creating ‘space for alternative approaches’. He also argued that the modern soldier should have ‘strong initiative and a questioning mind’, and that low-ranking commanders who were able to think of better solutions had ‘an equal responsibility to highlight this to the higher command’ [58]. 10 years later, as the SAF was completing its transformation into its so-called Third Generation (3G) Army, another article laid out the case for a thinking soldier. The authors opined that ‘[s]uccess or defeat in future battlefields will be determined by abilities possessed by individual soldiers on the ground … The “Thinking Soldier” will be equipped with cognitive tools to independently adapt to complex situations and make timely and right decisions in order to overcome local challenges whilst under extreme duress’ [59].

An increasing emphasis on the value of the individual soldier was also evidenced by the SAF’s initiative to transform the army from the Second Generation (2G) into a 3G force. On a broad scale, this was about the transformation of the SAF into an ‘integrated’ multi-service force that would be ‘flexible’, ‘leaner, better networked, more agile and lethal’, with the capacity to undertake a wide spectrum of operations. Part of being ‘better networked’ meant that the force would also turn to ‘technology as a force multiplier’ and that each soldier would be equipped with sophisticated data and network technology that would enable him or her to fight in the urban areas with ‘heightened situation awareness and precise engagement with hostile forces’. Known as the Advanced Combat Man System (ACMS), which was made up of different sensors, a mounted camera, a portable computer and a display attached to the soldier’s helmet, it became the epitome of what was envisioned as a thinking, technologically-advanced 3G soldier [59,60].

Laboratory-based exercise science research in the Soldier Performance Centre, which had already been established by Fabian Lim (the first SAF exercise physiologist) in the early 1990s, was able to meet the SAF’s research needs in this transformation. One major SPC tool that was well-utilised for this transformation was Singapore’s first climatic chamber, which had been built from scratch as a result of the SAF’s drive to reduce heat injuries in the 1990s. The chamber cost $1.2 million—an expensive item for its time—but it enabled the SAF, according to Lim, to be ‘more intelligent’ in soldier equipment and exercise prescription, such as by formulating fresh operational protocols to work with the newly-purchased submarine escape suits originally designed for cold conditions, as well as conducting investigations on combat load. The army, in particular, used the chamber for research on the equipping of the 3G soldier with the ACMS system, studying the effects of the extra combat load imposed on the soldier by technological wearables in a simulated hot-humid climate [49,61,62]. These efforts continued under the Defence Medical and Environmental Research Institute in the early 2000s, after Lim’s Human Performance branch in the SPC was shifted and integrated into the newly-founded DMERI.

### 4.3. New Standards

The SAF’s new emphasis on the value of the individual soldier was most pointed in the area of safety. While ‘a culture of safety awareness with its attendant risk assessment management systems … [had] steadily taken root throughout the organisation’ since the 1990s, it was the increasing public unacceptability of the training-related deaths of NS men and its relationship to safety that garnered the most attention since the 2000s. As a senior SAF Medical Officer puts it, the ‘risk calculus’ for the state pertaining to the acceptability of such deaths had shifted [55,63].

One of the first training-related deaths that captured public attention in the 2000s was the death of NS Second Sergeant Hu Enhuai through ill-treatment in the Combat Survival Course in 2003. Hu, along with other trainees, had their heads forcefully submerged in water. As a result, Hu died of asphyxia and near-drowning. In his statement to Parliament, Teo Chee Hean, then Minister of Defence, promised full public accountability and immediate action against the abusers. He concluded with a statement on what soldier safety meant:

‘Parents entrust their sons to MINDEF [Ministry of Defence] and the SAF to prepare and train them to perform a national duty in the defence of Singapore. This is a heavy responsibility that MINDEF and the SAF do not take lightly. We should not let up on hard and realistic training. But we will never compromise on safety nor risk the lives of our servicemen. It is MINDEF and the SAF’s solemn commitment to take in our young men, train them well and return them safely at the end … to their families as operationally-ready soldiers’ [64].

In a way, Teo’s statement set the bar for the way future training-related deaths were handled by the state. To be sure, Teo attempted to assert that rigorous training was important for the SAF. But his statements on safety were framed in absolute terms. That can be observed through his phrases ‘never compromise’ and ‘solemn commitment’. Therefore, preserving the lives of NS soldiers during peacetime training and ensuring their safe return to their families at the end of conscription was of paramount concern. What this suggests is that the state begun to view safety—the preservation of the lives of individual NS men during training—as a greater priority than training and performance.

It should be noted that Teo’s statement represents a shift from earlier attitudes towards training-related injuries in the 1980s—before the occurrence of a comprehensive state-driven emphasis on training safety in the late 1990s [56]. While safety was given substantial consideration then, the maintenance of training standards was accorded higher priority, implicitly signalling the state’s confidence in the safety guidelines that were being put in place, as well as a lack of concern over parental demands (likely due to their parliamentary dominance). In a response to a parliamentary question on flying training safety in 1984, then Minister of Defence Goh Chok Tong emphasised the importance of maintaining training standards while taking safety into account. He stated that the ‘greatest care’ had to be implemented to ensure that servicemen ‘are not pushed accidentally beyond the limits’, but ‘this must not be done at the expense of training standards’ [65]. In 1986, Lee Hsien Loong, who was then Minister of State for Defence, also stressed the importance of high training standards in the context of incorporating safety measures. He explained that soldiers ‘fall down … break bones … sometimes suffer heat exhaustion … [and] meet all kinds of mishaps despite all the precautions which are taken in the process of giving them realistic and tough training’ [66].

### 4.4. Soldier Safety-Shaped Measures and Research-Driven Measures

The state’s promotion of the soldier safety agenda shaped both the immediate measures that were put in place to deal with serious training-related accidents and deaths, as well as the medical fraternity’s research-driven measures. These soldier-safety shaped measures and research-driven measures were introduced from 2008 to 2018 (and beyond) in response to training deaths due to EHS and other causes, as well as emerging thermal concerns. Two initiatives which can be classified as soldier-safety shaped measures were the state’s introduction of a training suspension period after the occurrence of serious training-related accidents, and its drive to enhance all aspects of the SAF’s safety system through the immediate adoption of proven clinical and research-based operational practices. These are not considered research-driven measures as the state played a direct role in expediting the institution of these measures to respond to various incidents.

Firstly, calling for suspension in training (‘timeout’) became the state’s immediate approach towards appeasing the public that they were serious about reflecting on the Forces’ safety system during the occurrence of every training death. This was first practiced in 2008, when two NS men died from SAF-related physical activities—one of whom died from EHS while training in Brunei. For these incidents, Teo assured the Parliament then that ‘there were no lapses in the training safety system’ [67,68]. Subsequently, two out of the four timeouts that were called in 2011, 2012, 2018, and 2019 were preceded by EHS cases or deaths. Ng Eng Hen, the current Minister of Defence who oversaw the 2012 timeout, affirmed and expanded on Teo’s prioritisation of soldier safety, putting it in stark personal terms, explaining that each ‘Singaporean son is precious and any injury or death in the SAF is one too many’. For injuries and deaths to be prevented, Ng added, all soldiers and their commanders had to adhere to the SAF’s training safety regulations [69,70,71,72,73].

A second initiative has been the state’s drive to enhance all aspects of the SAF’s safety system with the immediate adoption of proven clinical and research-based operational practices after the occurrence of a serious training-related accident. One such example was especially taken in response to the death of Corporal First Class Dave Lee from EHS in April 2018, after he completed an eight km fast-march in training. Lee’s death was particularly jarring as he was misdiagnosed by the on-site medic as having ‘physical exhaustion’ and not heat injury, despite showing signs of the ailment. This led to ‘inadequate on-site casualty management and delayed evacuation to the medical centre’ where Lee was found to have a rectal temperature of 42.7 °C (a key clinical indicator of EHS is a rectal or core body temperature of >40 °C), and was diagnosed with EHS by the medical officer. This meant that the on-site treatment failed to cool Lee down and halt the progression towards EHS. Full-body cooling measures were immediately instituted but Lee’s condition did not improve. He eventually died of multi-organ failure after being warded for 12 days at the Changi General Hospital Intensive Care Unit [74]. A five-member external panel was called to review the SAF’s heat stress management system. The panel—while affirming that the system was at least as rigorous as that of the US, United Kingdom, Australia, and the North Atlantic Treaty Organization’s military forces—made direct recommendations which addressed the misdiagnosis, time delay and inadequate treatment that occurred in the case of Dave Lee’s EHS episode, as well as enhancements to the prevention regime. In terms of treatment, for instance, the panel recommended the introduction and use of the simple AVPU scale over the Glasgow Coma Scale as a key assessment tool to help medics and commanders recognise the signs of heat injuries. The AVPU scale (Alert, Response to Verbal Stimuli, Response to Pain Stimuli and Unresponsive) is taught to healthcare professionals and first aid providers for assessing a casualty’s level of consciousness. While the Glasgow Coma System is the ‘gold’ standard, it is ‘challenging to apply and time consuming’ [30,75].

For preventive measures, they also recommended the ‘full-scale implementation’ of ‘during-activity cooling’ methods (i.e., arm immersion in cool water) as a way to attenuate the internal heat stress of the soldiers during physical exertion. This cooling method had been successfully studied, used and implemented in the US Army. As such, the arm immersion measure was immediately adopted in 2018 [30,76,77,78].

On a longer-term basis, safety has been adopted as the eighth SAF Core Value (the official value system upon which the organisation is grounded) and a key performance indicator in terms of all forms of training-related thinking and operations. This has included a top-down promotion and enforcement of safety values and practices from the Minister of Defence to ground-level commanders and soldiers, as well as more efforts to empower ground-level soldiers as active agents in safety enforcement [75]. Major-General Goh Si Hou, who was Chief of Army from 2018 to 2022, for instance, articulated his ‘six core ideas’ about safety as a core value and performance indicator about a year after the death of Dave Lee. Some of his ideas included: (a) making safety a ‘mission outcome’ that is ‘in-built in all aspects of … training and evaluation’; (b) setting aside time to do each activity ‘well and safely’; (c) a ‘zero accident mindset’; and (d) the education and empowerment of every soldier to ‘take ownership’ of ‘safety outcomes’ [79].

According to Colonel (Dr) Lo Hong Yee, who was Chief of Medical Corps from 2019 to 2022, this has meant that the pursuit of safety in the SAF has been ‘relentless’ and that the ‘balance for the foreseeable future has shifted to safety’, over issues like the maximisation of physical performance. To him, the focus on safety is ‘not mutually exclusive’ with a focus on performance. Instead, it is fundamental to creating an armed forces that protects the lives of their soldiers in peacetime training and thereby gains the trust of the citizenry who entrust their sons to them. This is a ‘virtuous cycle’ that will help the SAF to better defend Singapore. Lo reveals that the top brass in the army pays extremely close attention to safety and medical data on injuries and deaths (in a sense, making safety a key outcome), and regularly issues advisories to manage the tempo of training at a macro level. Goh’s ideas, he explains, have also been incorporated into many army policies, such as making safety the basis of the ‘mission of defending Singapore’ and also allowing for the spreading out of training in order to ensure that army units and soldiers would not take unnecessary risks that could precipitate unwanted heat disorders or injuries [75].

In tandem with these timeouts and measures has been the intense public scrutiny over each training-related death during the 2010s. This has been reflected in a debate about keeping training both safe and ‘realistic’ (or ‘tough’), as well as a strong public response for the SAF’s full transparency in reporting about their investigations into these incidences. Three specific incidents—Private Dominique Sarron Lee’s death due to inhalation of smoke grenade fumes in 2012, Dave Lee’s death from EHS in April 2018, and Corporal First Class Liu Kai’s death from being crushed by an armoured vehicle—fuelled strong reactions from citizens. Hundreds turned up for the funerals of Dave Lee and Liu Kai, indicating intense public concern about the incidents [80,81]. Parents of some of the victims were particularly vocal about their need for the SAF to provide satisfactory answers as a form of accountability to them and Singaporeans. Dave Lee’s mother called for the SAF to implement ‘all possible measures’ against EHS while reminding commanders that while they had to train their sons to defeat enemies, it was their utmost responsibility to ‘return [the soldiers] home safe and sound to their loved ones’ [80]. An article asked the SAF to be transparent and timely in their reporting process each time a serious training-related accident occurred. This was to assure parents that they were ready to accept full responsibility, learn from the accidents, and mitigate all-possible risks to prevent future occurrences [82]. These reactions have encouraged the SAF leadership to affirm soldier safety as a response to these concerns. Dave Lee’s death, for instance, prompted Goh Si Hou to ask all army commanders to demonstrate through ‘actions’ that they stressed ‘utmost importance on training safety’ and ‘care for our soldiers’ [83]. While a commentary raised the question of whether ‘soft’ training or training which emphasised safety over performance would affect the viability of defending Singapore, it is evident that soldier safety has become a dominant principle which has reshaped SAF training and supplied the organisation with a means to address parental demands [84].

This resolute commitment to soldier safety can be best exemplified by Ng Eng Hen’s conclusion to his 2019 parliamentary speech after the training-related death of another NS soldier:

‘Over the past 17 months, four national servicemen have passed away during training. In 2012, the SAF also experienced four training fatalities that year. But from 2013 to 2016, we had none. How did this turnaround occur? This was probably due to multiple factors, but I think the new safety measures we put into place after the devastating incidents of 2012 had an effect …’

‘We must never give up on National Service that forms the backbone of our SAF for national defence. This imperative of National Service and our national defence does not absolve … the accountability of MINDEF and the SAF in any way, to ensure safe training. On the contrary, it compels MINDEF and the SAF to do all that is humanly possible to prevent training deaths for national servicemen because precious sons have been entrusted to us by their families. MINDEF and the SAF will hold ourselves accountable for every single national serviceman entrusted to us. I am deeply sorry for the loss of four precious national servicemen in the last 17 months. The SAF will strengthen its safety systems. Even as we honour those who died in service of our country, we must soldier on to build a strong defence, to protect Singapore and to protect every national serviceman during training’ [85].

This resolve was also seen in the state’s influence on the medical fraternity’s research-driven measures. Soldier safety-shaped research-driven measures refers to the state’s imprinting of the soldier safety agenda on the direction of heat stress research matters, and the internalisation of this agenda in the medical fraternity’s research. A key example of such imprinting was the state’s exertion of the soldier-safety logic on heat acclimatisation (HA) in training. Although the SAF had already incorporated the principles of HA through their scientifically-based progressive training for recruits since the 1980s, their decision to equip the soldiers with a new bullet-proof body armour vest in the late 2000s sparked fresh thermal concerns, which resulted in their commissioning of DMERI to study HA as a specific mitigative measure for the heat load induced by the body armour. Jason Lee, one of the early Exercise Science Ph.D. holders who was employed by DMERI, was the Principal Investigator of the HA research project [86]. One of the key findings of Jason Lee’s study was that HA did not produce a significant drop in core temperature—one of the crucial physiological indicators of increased tolerance to thermal stress—after soldiers underwent a 10-day HA regime. While this was not to say that there was no acclimatisation effect, what it meant was that acclimating human beings who are used to a hot-humid environment can only produce some mild gains when training in a similar climate. Nonetheless, from our knowledge, the SAF’s urgent drive to introduce the body armour meant that they were willing to implement the marginal physiological benefits from the proposed 10-day HA regime and craft a public narrative which explained that the body armour was necessary for saving lives in the battlefield, and that the HA programme was an added mitigation to reducing the heat strain generated from it [87]. This position was conveyed differently by the medical fraternity two years later when they explained that HA was part of a series of measures to help recruits adapt to physical exertion in a hot environment due to their largely sedentary, ‘air-conditioned lifestyle’ [8]. Overall, the coupling of the two issues, despite knowledge that the benefits were marginal, was demonstrative of how the state’s soldier safety agenda became the driving factor for the implementation of this new HA regime. In this case, the SAF leadership was keen to use this new HA programme to assure the public that it was treating soldier safety with utmost importance.

The state’s imprinting of the soldier safety agenda on the medical fraternity’s research work can also be seen in the area of cooling measures. In a bid to respond to the occurrences of EHS from 2012 to 2014 (see Table 1), and the aftermath of Dave Lee’s EHS death in 2018, the state influenced the adoption of new preventive and therapeutic cooling methods for heat exhaustion and EHS victims in order to show that they were enhancing the SAF’s heat stress management capabilities. From 2013 to 2016, they commissioned the medical fraternity to study the efficacy of a commercial cooling pad solution and portable cold-water immersion system. Both methods were found to have cooling rates superior to those of the BCU on hyperthermic subjects [30,88]. Due to its cooling rate and portability, the commercial cooling pad solution was implemented as an on-site cooling method, serving as a superior alternative to the use of ice packs. The aftermath of Dave Lee’s EHS in 2018 also prompted the state to embark on another cycle of enhancing their heat stress management cooling regime. This turned their attention to the potential of prophylactic cooling measures to enhance the heat tolerance levels of their soldiers. Here, we observe that the state’s emphasis on the soldier safety agenda was more of a co-optation of the best-available local research. As we have seen, the state followed the recommendations of the review panel and immediately instituted arm immersion as a cooling measure. On top of this, based on our knowledge, they also turned to Jason Lee, a member of the medical fraternity who had been researching on the ingestion of cold drinks as a form a pre-exercise and exercise-based cooling aid since 2004. Independent studies from DSO and other research groups showed promising results which demonstrated that ice-slurry ingestion increased the thermal tolerance of individual soldiers. The favourable results, as well as the impetus from the soldier safety agenda, have impelled the SAF leadership to accept and gradually implement ice-slurry as a pre-cooling and during-exercise cooling measure for their training since 2022. Ice slurry provided an appropriate soldier-safety response to enhancing the preventive cooling capacity of the SAF. This measure was initially used for selected, specialised groups such as the commandos, before its recent implementation to other vocations within the SAF [89,90,91,92].

In spite of the state’s heavy-handedness, we should note that the medical fraternity did not merely passively wait for the state’s directions, but also actively responded to the state’s key concerns by internalising the soldier safety agenda in their research-driven measures. Two recent projects illustrate how they did so. They are the SAF thermoregulatory test, and the profiling of commando trainees for their signature 72 km graduation route march. Firstly, the SAF thermoregulatory test is used as a tool to determine the return to service of personnel with a history of EHS. Drawing on evidence-based testing conducted in other armies as well as local research, this test is an objective tool that is used to assess the thermoregulatory function of EHS casualties, ensuring the safe return of heat injured personnel to combat duty [93]. Specifically, this test was also introduced as a form of return to duty (and ‘recourse’) for the SAF’s professional soldiers who were monitored for their heat tolerance thresholds during their training and operations in order to determine whether there were ‘deficiencies’ in their thermoregulatory function. Such monitoring was a preventive measure, and can be considered as an initiative which internalised the soldier safety agenda. This is because soldiers who were deemed as deficient through the monitoring sessions would be asked to step down from full-scale training and operations, in consideration of their higher risk of attaining an exertional heat injury through such activities. As such, the implementation of the SAF thermoregulatory test served as a tool for return to duty for these professional soldiers. That is, taking and passing the test after being identified as deficient in thermoregulatory function is a requirement in allowing them to return to full-scale training and operations [55]. In other words, the test would be a demonstration that they were sufficiently safe for training by acquiring a level of thermoregulatory tolerance to mitigate their deficiency, and thereby, the risk of heat injury.

Another significant study was the profiling of commando trainees (many of whom are NS men) who undertook the 72 km graduation route march at the end of their course—meant to be a highly-demanding training activity. Again, such profiling was driven by soldier safety concerns. Surprisingly, the profiling yielded lower than expected thermal and physiological strain among participants. This resulted in a revision of standards to make the graduate route march more challenging. This case is a good example of how research arising from safety concerns also has implications for the performance standards of the SAF’s soldiers. It suggests that there is a need to keep a balanced view on both safety and performance aspects by understanding what the lower and upper limits of exertional heat stress are [94]. Beyond those projects, the medical fraternity also initiated various research studies in the recent years with soldier safety as a core consideration. Some of these include the formulation of a work-rest cycle for soldiers who work in fully-encapsulated environments (i.e., full protective suits), a review of the SAF hydration regime in terms of whether it can be tailored according to exercise intensity levels, and a novel weather-based study which examined the use of various environmental indicators as a predictor of heat injury risk. The results of the latter study have already been translated into usage as a predictive tool in the SAF [55,95]. As can be seen, these initiatives internalised the soldier safety agenda and sought to enhance existing research-driven measures which have been in place to manage heat disorders, as well as to produce new heat stress management tools for the SAF.

While most of the soldier-safety shaped measures and research-driven measures were implemented as part of short-term reactions to serious training-related accidents and emerging thermal concerns, it can be argued that they have played a role in maintaining the SAF’s low heat injury incidence rate in the 2000s and 2010s (Table 1). Nonetheless, the short-term and response-driven nature of such research work has limited the medical fraternity to ad-hoc enhancements of the research-driven measures which have been instituted since the 1980s. Significantly, though these enhancements have likely contributed to the maintenance of a low heat injury rate, they do not provide plausible pathways to solving the state’s ultimate concern of eradicating all training-related deaths, especially in relation to EHS.

### 4.5. Implications

The incorporation of the soldier safety agenda has broader implications for heat stress management. Recent studies on heat injury management in the US military underscore that leadership is a crucial factor in mitigating the risk of heat injuries, especially at the organisational level [6,96]. In this respect, the SAF’s history of heat health management can be interpreted as a model example of leadership, especially in terms of both the state’s and the medical fraternity’s drive to incorporate soldier safety into its heat mitigation mindset and strategies. That said, we should note that the US military empowers its ground commanders to be part of the process of heat injury mitigation by equipping them with a five-step operational risk management process which serves as a decision-making tool. This process, which involves the identification of hazards and the institution of controls against these hazards, is used to determine the level of heat stress risk for training and operations, and to allow ground commanders to make specific controls and adjustments on the basis of the assessed risks in their contexts [6,96]. Such empowerment has not been observed in the case of the SAF, and it is proposed that similar steps can be taken to enable the SAF’s ground commanders to become directly involved in the work of heat injury mitigation identification, assessment and evaluation. This could further aid the internalisation of the soldier safety agenda at the level of local ground units. The incorporation of such leadership tools in the SAF can also be potentially translated to the sports and outdoor worker industries through inter-agency cooperation [96]. Best practices in equipping local-level leaders with heat mitigation decision-making tools in the military should have a direct influence on different sectors of the civilian community which face similar challenges.

## 5. Conclusions

**Figure 2:**
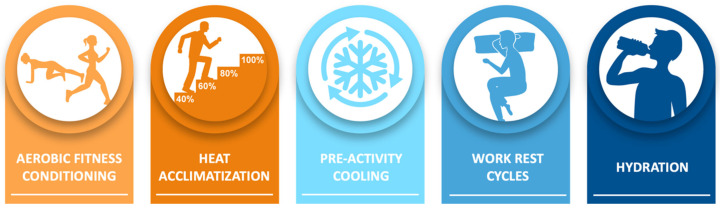
Holistic and Evidence-Based Interventions in Reducing Heat Injury and Improving Performance in the SAF [97].

This is the first study which examines how the changing relationship between the state and military medical fraternity shaped the implementation of heat stress management policies for a largely conscript-reliant Singapore Armed Forces from the late 1970s to the present day. Our findings show that the fundamental basis of the relationship between the state and medical fraternity did not change, but the power dynamics of the relationship shifted due to changing contexts. Research-driven scientific measures have been the main pillar for the SAF’s heat stress management policies. As we have seen, the state has been consistently supportive of the medical fraternity’s research-driven measures. By allowing the medical fraternity the leeway and autonomy to craft their local research and establish institutions like the Soldier Performance Centre to aid them in such work, they became the main shapers of heat stress management policies and played a key role in crafting and implementing a range of measures which have solved the heat injury crisis and reduced the number of exertion-related heat disorders to an extremely low rate since the 2000s. The emergence of new contexts, such as the new normal of low rates of heat injuries and the recognition of the significance of every individual soldier brought about the state’s concern in soldier safety. The new concern altered the way in which the state saw its role in relation to the fundamental pillar of research-driven measures. Under this new soldier safety agenda, it decided to play a more activist role to influence the direction of these research-driven measures. This included expediting the implementation of measures which were already proven clinically and had a research-basis, and imprinting the soldier safety agenda on the direction of the medical fraternity’s research in areas which required responses to emerging thermal concerns and serious training-related EHS accidents. The medical fraternity also internalised this agenda and launched ad-hoc enhancements of various research-driven measures. That said, despite the state’s heavy-handedness in promoting soldier safety, the medical fraternity held on to its research autonomy. Overall, it should be acknowledged that the ad-hoc enhancements and additions to the suite of research-driven measures (Figure 2) have played a role in maintaining the low incidence of all types of heat injuries in the SAF.

As pointed out, a limitation which has emerged because of this shift towards soldier safety is that the medical fraternity’s research work has been guided by short-term cycles of attention and reaction to adverse events and state-based concerns. This has constrained the medical fraternity’s capacity to explore research areas that could plausibly supply answers to the state’s ultimate concern of eradicating all training-related deaths (in this case, deaths related to EHS). A potentially fruitful avenue of research which can transcend these short-term cycles is the individualisation of technological capabilities in heat stress prevention and treatment that go beyond the current state of technology. Exploration in this area would address the concerns raised by soldier safety, and make key goals like the potential eradication of all training-related deaths as core research questions that need to be addressed by the medical fraternity.

Climate change in Singapore and across the world is a long-range problem of existential nature that could serve as a possible impetus for the medical fraternity to transcend the short-term cycles and shift towards research on core issues that can only be addressed under a long-term research plan. The increased sophistication and usage of digital technologies like artificial intelligence and instant messaging platforms has also made technological practices like the remote monitoring of individuals more viable. This can be seen in the rapid integration of instant digital monitoring technologies in popular food delivery and logistical applications, as well as the widespread global uptake of telecommunication software as tools of everyday working and living during the COVID-19 pandemic. This has produced a culture which is attuned to, and expects real-time feedback through, such technological products—a condition which had not emerged in such a widespread fashion during the 2000s and the early 2010s [55]. This suggests that this may be the opportune time for the medical fraternity to make serious investments in technologies for individualisation (particularly, in technologies like personalised heat health monitoring or more generally, remote physiological monitoring with an emphasis on heat stress) in order to address pressing concerns about the effect of anthropogenic-driven climate change on the SAF’s training, as well as to develop plausible solutions to the goal of eradicating training-related EHS deaths. This paper concludes by providing two reflections on these aforementioned issues:

Firstly, the potential of technologies for individualisation to collect and generate individual- and population-based physiological data for heat stress management interventions can help the SAF to transcend the short-term cycles. On one level, the collection of data on the individual level would enable the formulation of individualised prevention and treatment solutions which are derived and tailored from the whole battery of research-driven measures. For instance, a fully-operational personalised heat health monitoring system can be employed to recognise the risk factors and causes of heat injury among individual soldiers through the real-time monitoring of their daily health status and physical condition. This could include ‘real-time’ reports on hydration status, sweat rates and responses, or status updates on certain biomarkers [96]. Climate change can also be accounted for in the real-time monitoring through the identification of it specific impacts (e.g., extreme weather events or rising yearly temperatures) on the health status and risk factors of individual soldiers. Personalised interventions can be identified and prescribed for the individual on the basis of these collated health status markers, risk factors, climatic factors and causes of heat injury from a personal heat health monitoring device. Specifically, such data could be trained on algorithms and artificial intelligence systems to identify the most suitable solutions to mitigate the risk factors. Additionally, the identification of causes would be useful for investigating and learning about specific causal pathways which lead to different types of heat injury. Such knowledge would be helpful in allowing the medical fraternity to embark on research which targets the eradication of all training-related EHS deaths. On another level, the individualised data can be aggregated as real-time, population-based datasets in order to identify broad-based heat injury risk factors, trends, climatic factors, and the overall health and fitness status of the SAF and its various sub-units and groups. In other words, the constant monitoring and collation of such data can form the basis of a renewed heat injury surveillance system which will be able to capture and analyse key trends, patterns and risks at both the micro and macro levels. Acquiring such knowledge could allow the SAF to adjust its organisation-wide measures to target specific heat injury risks and trends, or to refine its training tempo and intensity to mitigate any change in population-based heat injury risks and risk factors. It should be noted that the state and medical fraternity have recently identified a pathway forward in this direction with the formation of the new Heat Resilience and Performance Centre—a collaboration between the SAF, DSO National Laboratories and the National University of Singapore. This centre, based within the School of Medicine at the National University of Singapore, will take a long-term approach to developing heat stress solutions for the SAF with soldier safety as a core principle (and its attendant concerns like the elimination of all training-related EHS deaths) that is inbuilt into its approach. This can be seen in the ‘key thrusts’ of the centre, which will mainly focus on developing individualised and organisational capabilities in surveillance, detection and prevention to mitigate the risks of heat injuries among the SAF’s servicemen and women [98].

Secondly, Singapore’s year-round hot-humid climate lends itself as a strong candidate for being a site which can provide a ‘rehearsed climate change or global warming scenario’ for testing technologies for individualisation. This can be coupled with the fact that Singapore is also experiencing the effects of global warming, as evidenced by the island’s increasing average mean temperature (26.9 °C in 1980 to 28.0 °C in 2020) [99]. Therefore, outcomes from such testing could be helpful for militaries and outdoor worker populations across the world who are being exposed to an increasing frequency of extreme-heat events. This exposure is widely acknowledged as a serious threat to the health of these individuals and communities, especially those who are marginalised and vulnerable. An example is the case of low-wage migrant outdoor workers who undertake long-term strenuous work under extreme heat conditions in the Middle East, Asia and Africa. Such work has resulted in the emergence of new heat disorders among these workers, largely to do with the organ damage of the kidneys and heart. Increasing rates of cardiovascular mortality have been observed among Nepalese migrant construction workers who worked in Qatar, especially during the hot summer months [100,101,102]. Within the last decade, the US Army has also recognised the emergence of this problem among their personnel. This led to the creation of a new category called ‘heat injury’ in their classification system for exertional heat illnesses. In this classification, heat injury refers to ‘heat exhaustion with evidence of organ (liver, renal, stomach) and/or muscle (rhabdomyolysis) damage, but without the neurological signs and symptoms of heat stroke’ [103]. If the SAF implements and operationalises the use of technologies like personalised heat health monitoring, it will be well-placed as an organisation to test for the prevalence of novel strains of heat disorders, such as the aforementioned strain of exertional heat exhaustion with organ damage. Currently, there is still a lack of understanding about this new heat injury strain. Thus, collecting real-time physiological and climatic data from heat-injured servicemen and women in the SAF could help to throw light on the extent to which physical activity in a perpetually warm climate could be linked to organ damage from heat exhaustion. Population-based physiological and climatic data collected from healthy and heat-injured SAF soldiers could also be used to detect and predict new strains of heat disorders that could emerge under extreme heat. This would allow the SAF to serve as a new model of heat injury detection, prevention and prediction for militaries and outdoor workers around the world.

## Figures and Tables

**Table 1:** Publicly Reported Exertional Heat Injuries in the SAF, 1971–2018 [9,27,28,29,30,31].

Year	Heat Stroke	Heat Exhaustion	All Heat Injuries	Deaths Due to EHS
1971	2	Not reported	Not reported	Not reported
1974	1	Not reported	Not reported	1
1975	1	Not reported	Not reported	1
1976	3	223	226	3
1977	2	210	212	2
1978	Not reported	Not reported	Not reported	2
1980	5	52	67	0
1981	0	32	56	0
1982	3	17	30	0
1983	16	15	97 [32]	0
1984	8	70	78 [32]	1
1985	25	94	119	Not reported
1986	46	119	165	Not reported
1987	32	208	240	Not reported
1988	32	220	252	Not reported
1989	22	159	181	Not reported
1990	10	114	124	Not reported
1993	Not reported	Not reported	Not reported	1
1994	Not reported	Not reported	Not reported	2 (1986-1994: 5 deaths) [33]
1996	Not reported	Not reported	80 (1990s: 200 per year) [33]	0
1997	Not reported	Not reported	69	0
2008	Not reported	Not reported	Not reported	1
2011	2 + 1?	Not reported	2000s: 20 per year [8]	1? [34]
2012	8	19	27	0
2013	3	30	33	0
2014	6	26	32	0
2015	0	24	24	0
2016	3	23	26	0
2017	5	15	20	0
2018	1	Not reported	Not reported	1
